# Genetic Diversity and Population Structure of the Secondary Symbiont of Tsetse Flies, *Sodalis glossinidius*, in Sleeping Sickness Foci in Cameroon

**DOI:** 10.1371/journal.pntd.0001281

**Published:** 2011-08-23

**Authors:** Oumarou Farikou, Sophie Thevenon, Flobert Njiokou, François Allal, Gérard Cuny, Anne Geiger

**Affiliations:** 1 UMR 177, IRD-CIRAD, CIRAD TA A-17/G, Campus International de Baillarguet, Montpellier, France; 2 Faculty of Science, University of Yaoundé I, Yaoundé, Cameroon; 3 CIRAD, UMR Trypanosomes, Montpellier, France; 4 CIRAD, UPR Diversité Génétique et Amélioration des Espèces Forestières, Montpellier, France; International Centre of Insect Physiology and Ecology, Kenya

## Abstract

**Background:**

Previous studies have shown substantial differences in *Sodalis glossinidius* and trypanosome infection rates between *Glossina palpalis palpalis* populations from two Cameroonian foci of human African trypanosomiasis (HAT), Bipindi and Campo. We hypothesized that the geographical isolation of the two foci may have induced independent evolution in the two areas, resulting in the diversification of symbiont genotypes.

**Methodology/Principal Findings:**

To test this hypothesis, we investigated the symbiont genetic structure using the allelic size variation at four specific microsatellite loci. Classical analysis of molecular variance (AMOVA) and differentiation statistics revealed that most of the genetic diversity was observed among individuals within populations and frequent haplotypes were shared between populations. The structure of genetic diversity varied at different geographical scales, with almost no differentiation within the Campo HAT focus and a low but significant differentiation between the Campo and Bipindi HAT foci.

**Conclusions/Significance:**

The data provided new information on the genetic diversity of the secondary symbiont population revealing mild structuring. Possible interactions between *S. glossinidius* subpopulations and *Glossina* species that could favor tsetse fly infections by a given trypanosome species should be further investigated.

## Author Summary

Human African trypanosomiasis remains a threat to the poorest people in Africa. The trypanosomes causing the disease are transmitted by tsetse flies. The drugs currently used are unsatisfactory: some are toxic and all are difficult to administer. Furthermore, drug resistance is increasing. Therefore, investigations for novel disease control strategies are urgently needed. Previous analyses showed the association between the presence of *Glossina* symbiont, *Sodalis glossinidius*, and the fly infection by trypanosomes in a south-western region in Cameroon: flies harbouring symbionts had a threefold higher probability of being infected by trypanosomes than flies devoid of symbionts. But the study also showed substantial differences in *S. glossinidius* and trypanosome infection rates between *Glossina* populations from two Cameroonian foci of sleeping sickness. We hypothesized that the geographical isolation of the two foci may have induced the independent evolution of each one, leading to the diversification of symbiont genotypes. Microsatellite markers were used and showed that genetic diversity structuring of *S. glossinidius* varies at different geographical scales with a low but significant differentiation between the Campo and Bipindi HAT foci. This encourages further work on interactions between *S. glossinidius* subpopulations and *Glossina* species that could favor tsetse fly infections by a given trypanosome species.

## Introduction

Tsetse flies are medically and agriculturally important vectors that transmit African trypanosomes, the causative agents of sleeping sickness in humans (human African trypanosomiasis – HAT) and Nagana in animals. This debilitating disease still affects a wide range of people in sub-Saharan Africa [1] and is invariably fatal if untreated. Nagana is estimated to cost African agriculture US$4.5 billion per year [2]. The drugs currently used are unsatisfactory: some are toxic and all are difficult to administer in humans [3]. Furthermore, drug resistance is increasing [4]. Therefore, investigations for novel drugs and/or novel disease control strategies are urgently needed.

The biological process leading to transmission of the trypanosomes from one mammalian host to another is complex. Prior to being transmitted, the parasite must first establish in the tsetse fly midgut following an infective blood meal. Then it must mature either in the salivary glands or in the mouthparts, depending on the trypanosome species [5], [6]. This ability to acquire the parasite, favor its maturation, and transmit it to a mammalian host is known as vector competence. It depends on both *Glossina* and trypanosome species.

Tsetse flies harbor three different symbiotic microorganisms [7]. One of them, *Sodalis glossinidius* [8], [9], a maternally transmitted secondary endosymbiont, is suspected of being involved in the vector competence of *Glossina* [10]–[12]. The reported full-length sequencing of the complete genome [13] and extrachromosomal DNA [14] showed that *S. glossinidius* displays active mechanisms of cellular interactions and is an intermediate between free-living and obligate intracellular bacteria evolving toward a specific interaction with *Glossina* [13], [14].

*G. palpalis gambiensis* (*palpalis* group) and *G. morsitans morsitans* (*morsitans* group) were shown to harbor genetically distinct populations of *S. glossinidius* [15]. Neutral evolution is likely to explain this result, but, interestingly, *G. palpalis gambiensis* and *G. morsitans morsitans* transmit preferentially different trypanosomes species. The ability of *Trypanosoma brucei gambiense* and *Trypanosoma brucei brucei* to establish in *G. palpalis gambiensis* midgut was further linked to the presence of *S. glossinidius*-specific genotypes in the insectarium [16]. This suggests that vector competence might be linked to given genotypes of *S. glossinidius* rather than a mere presence/absence of the symbiont.

Given that flies multiplying in the insectary may undergo specific selective pressures that differ from those in the natural environment, the possibility that field environmental conditions of HAT foci may lead to alternative results could not be excluded. Therefore, an epidemiological investigation was conducted in two HAT foci in the south of Cameroon. This study took into account a large panel of criteria to test the existence of interactions between the three *Glossina*-trypanosome-symbiont partners. The results showed that the trypanosome infection was not randomly distributed between subpopulations of field flies harboring *S. glossinidius* or free of *S. glossinidius*. Statistical analyses confirmed the association between the presence of the symbiont and the field flies' infection by trypanosomes: the parasite prevalence was nearly threefold higher within the populations of flies harbouring the symbiont than within those that did not harbour the symbiont [17].

The results obtained from fly trapping, the prevalence of *S. glossinidius* and trypanosomes, and the different symbiont/parasite associations also showed significant differences between the fly populations from the two HAT foci.

We hypothesized that the geographical isolation of the two foci may have induced independent evolution of fly and symbiont populations in each area, resulting in a diversification of symbiont genotypes. If so, it may be assumed that such genotypes may interact differently with *Glossina* species and may favor fly infection by a given trypanosome species in different ways.

The foregoing assumption necessitated a large-scale analysis of the genetic diversity of *S. glossinidius* and the distribution of the different genotypes within the fly populations of the different sampling areas. Following preliminary assays, microsatellite markers seemed to be well suited to perform this analysis.

To the best of our knowledge, the present study is the first large-scale genetic population investigation on the tsetse fly symbiont, *S. glossinidius*, from *Glossina palpalis palpalis* flies sampled in Campo and Bipindi, two sleeping sickness foci in Cameroon. The aim was to analyze their genetic diversity in order to determine the genetic structure of *Sodalis* and to study possible rates of gene flow at various spatial scales of Cameroonian *S. glossinidius* populations.

## Methods

### Collection of *Sodalis glossinidius*

*Glossina palpalis palpalis* flies were collected in two HAT foci (Bipindi and Campo) situated in the Ocean Division of the South Region of Cameroon. The Campo focus (2°20′N, 9°52′E) presents several biotopes (farmland, marshes, swampy areas, and equatorial forest). The Bipindi focus (3°2′N, 10°22′E) has a typical forest bioecological environment including equatorial forest and farmland along roads and around villages. Both foci contain highly diversified wild fauna [18], [19].

The Bipindi focus has been known since 1920 [20] and is still active since 60 new patients were detected between 1998 and 2002 [20] and two in 2006–2007 (V. Ebo'o Eyenga, pers. comm.). Bipindi covers several villages, mainly located along roads. It is surrounded by hills and has a dense network of rivers crossing cocoa farms, offering suitable habitats for tsetse flies.

Campo is located on the Atlantic coast and extends along the Ntem River [21]. It is characterized by an equatorial rain forest zone with a network of several rivers, swampy areas, and marshes. During the epidemiological survey conducted in 2006–2007, ten cases of sleeping sickness were detected (V. Ebo'o Eyenga, pers. comm.). The two HAT foci (Bipindi and Campo) are located on different river basins.

Entomological surveys were conducted in 2007 in Bipindi and Campo. The geographical positions of the sampling sites were determined using the global positioning system. Tsetse flies were captured using pyramidal traps [22] planted in suitable tsetse fly biotopes. Each trap remained deployed for four consecutive days and flies were harvested twice a day. The different *Glossina* species were first identified and then sorted into teneral and non-teneral flies, according to morphological criteria [23]. Tenerals are young flies that have never taken a blood meal and that have never got the opportunity to get infected by trypanosomes; thus they were discarded from the experiment design. The non-teneral flies were dissected in a drop of sterile 0.9% saline solution, and their midgut separately transferred into microfuge tubes containing ethanol (95°) for further symbiont analyses. The instruments used were carefully cleaned after the dissection of each fly to prevent contamination. During field manipulations, the microfuge tubes were maintained at room temperature; thereafter, they were stored in the laboratory at −20°C until use.

### DNA extraction

DNA was extracted from tsetse fly midguts using cetyl trimethyl ammonium bromide (CTAB) as described by Navajas et al. [24] and processed according to the previously published methodological report [25]. Briefly, tissues were homogenized with a pestle in a CTAB buffer (CTAB 2%; 0.1 M Tris, pH 8; 0.02 M EDTA pH 8; 1.4 M NaCl) and incubated at 60°C for 30 min. The DNA was extracted from the lysis mixture with chloroform/isoamylic alcohol (24/1; V/V) and precipitated by adding isopropanol (V/V) to the DNA containing phase. After centrifugation (10,000 *g*, 15 min), the pellet was rinsed with 70% ethanol, air-dried, and resuspended in distilled sterile water. The DNA samples were stored at −20°C until PCR amplification.

### Selection of microsatellites in the *Sodalis glossinidius* genome and primer design

This step was processed as in Farikou et al. [25], with however several modifications. We used the three microsatellites (ADNg 5/2; ADNg 21/22 and ADNg 15/16) described in Farikou et al. [25]. Besides, we amplified a fourth microsatellite (ADNg 12/13) from the sequence published in Genbank (GenBank accession number AP008232), according to its potentiality to generate high level of polymorphism [26]; its primers were designed using the software Primer3 (http://frodo.wi.mit.edu/primer3/) (Table 1, Table 2).

**Table 1:** *Sodalis glossinidius* microsatellite markers, PCR primers, and allelic polymorphism.

Marker	Repeat sequence^a^	Primer sequences (5′-3′)	Location at bp^b^	*Na*	Size range of alleles (bp)
ADNg 12/13	(GC)×7	1 TGCCAGCCGCTCGATAAGG	3399960–3400122	3	159–165
		GGTATTACCCAATCAAATCGTG			
ADNg 5/2	(AC)×7	GGCCGGTATTCTAACCGAC	4115043–4115222	4	174–180
		2 AACTGCCAGGCATCCATTAC			
ADNg 21/22	(GCC)×9	1 GAGCAAATCTCCCAGCACAT	1450588–1450759	6	163–178
		TTCTTGTCCCTCAACCCATC			
ADNg 15/16	(AGG)×11	1 ATACGGCGAAGCAATGAGAC	3250160–3250283	6	103–118
		CAGCCTCTAAGCGCTCAACTC			

a Number of nucleotide repeats as published in GenBank for the corresponding strain.

b GenBank accession number AP008232.

*Na*, number of alleles.

1 Primer labelled with infrared dye (IRD) IRD800.

2 Primer labelled with infrared dye (IRD) IRD700.

**Table 2:** Summary of the characteristics of the developed primers.

Marker	Primer sequences (5′-3′)	% GC	MW (g/mol)	Tm (°C)	Ext. coeff (l.mol^−1^.cm^−1^)
ADNg 12	TGCCAGCCGCTCGATAAGG	63	5813.82	62	179100
ADNg 13	GGTATTACCCAATCAAATCGTG	41	6718.45	62	218000
ADNg 5	GGCCGGTATTCTAACCGAC	58	5788.81	60	179400
ADNg 2	AACTGCCAGGCATCCATTAC	50	6045.99	60	189900
ADNg 21	GAGCAAATCTCCCAGCACAT	50	6055	60	193600
ADNg 22	TTCTTGTCCCTCAACCCATC	50	5938.9	60	170900
ADNg 15	ATACGGCGAAGCAATGAGAC	50	6184.1	60	208400
ADNg 16	CAGCCTCTAAGCGCTCAACTC	57	6311.14	66	188500

For each of the four couples of primers, one of the primers was 5′ end labelled with an infrared dye (IRD700 or IRD800) for sizing the PCR products with an automatic sequencer. Primers were synthesized by MWG (Ebersberg, Germany).

### DNA amplification

Specific primers amplifying tsetse fly mitochondrial DNA were used to control the quality of the extracted DNA, as previously described [8]. Specific polymerase chain reaction (PCR) detection of *S. glossinidius* was performed on midgut-extracted DNA, as previously described [17]. Midguts showing specific detection of *S. glossinidius* were further processed for *S. glossinidius* microsatellite genotyping. The method was adapted from Farikou et al. [25].

The amplification reaction mixture consisted of 10 mM Tris–HCl (pH 8.3), 50 mM KCl, 1.5 mM MgCl_2_, 0.2 mM dNTPs (QBiogene), 4 pmol of each primer, 0.6 U *Taq* DNA polymerase (QBiogene), and 3 µl of fivefold diluted DNA in a 20-µl reaction volume. Amplifications were carried out as follows: 3 min at 94°C for initial denaturation, 35 cycles of denaturation at 94°C, annealing at 58°C for the marker ADNg5/2 and 55°C for the markers ADNg15/16, ADNg12/13, and ADNg21/22, and extension at 72°C for 30 s. The final cycle was followed by an additional 10 min at 72°C to complete polymerization.

Primer sets for each locus were tested to ensure that they specifically amplified *S. glossinidius*, and not host (fly) DNA. In order to assess whether the amplicons corresponded to *S. glossinidius* microsatellites and to determine the number of microsatellite repeats, the different alleles were cloned into PGEM-T Easy (Promega, Charbonnières, France). For each different allele, one recombinant plasmid was then sequenced (GenBank accession numbers JN032317–JN032335) and compared with the reference sequence of *S. glossinidius* [13] (GenBank accession number AP008232). The number of the repeat elements was determined by sequence analysis. Negative controls, consisting of extraction reagents without tsetse fly material, were used throughout the isolation procedures and included in PCR assays along with several template blanks (water) to ensure the absence of contamination in typing experiments.

### Electrophoresis of PCR products

After specific amplification, infrared dye-labeled (IRD700 or IRD 800) PCR products were diluted to 1/5, 1/10, or 1/50 in loading buffer (95% deionized formamide, 20 mM ethylenediaminetetraacetic acid (EDTA), pH 8.0, and 1 mg/ml bromophenol blue). Then they were denatured for 3 min at 95°C, and transferred to ice before loading. The sample-loading volume was 1.22 µl. Each mixture was separated, in a 1- to 2-h run at 1500 V, on a 6.5% (wt/vol) Long Ranger polyacrylamide gel, using 1× Tris-borate-EDTA buffer (Bio-Rad, Hercules, CA, USA), on a two-dye, model 4300 LI-COR-automated DNA sequencer.

Infrared images of the patterns were analyzed using the semiautomated scoring program Quantar (version 1.05; KeyGene products B.V., Wageningen, The Netherlands). Measurement of allele length on polyacrylamide gels was automated using molecular size markers.

### Statistical analyses

#### Genetic diversity estimation

Finally, genetic diversity was analyzed on 244 samples carrying the symbiont. Of these, 131 were from the Campo focus, corresponding to three villages: 38 from Akak village (N 02°22.831′, E 09°58.654′), 33 from Campo Beach/Ipono (N 02°20.985′, E 09°50.300′), and 60 from Mabiogo (N 02°17.657′, E 09°51.938′). 113 samples were from the Bipindi focus: Ebimimbang village (N 03°02.856′, E 10°28.515′).

The populations corresponded to the four villages. The mean number of alleles per locus, the allele frequencies, and the level of heterozygosity (*H*_E_) were estimated for each locus and each population using ARLEQUIN software, version 3.5.1 [27]. Microsatellite alleles were then combined into haplotypes to perform the following analyses. Each population was characterized by its level of diversity using the number of detected haplotypes, the haplotypic diversity (*H*_Eh_) [28], and the rarefied haplotypic richness (*H_R_*), computed using the CONTRIB 1.02 program [29]. The rarefied haplotypic richness (*H_R_*) is defined as the expected number of different haplotypes found in each population using a standardized sample size fixed as the smallest available number of genotyped individuals.

#### Genetic structure

The genetic structure among *S. glossinidius* populations, at the village level and the HAT foci, was tested using the analysis of molecular variance (AMOVA, ARLEQUIN) based on haplotype frequencies [30]. AMOVA subdivided the genetic diversity into hierarchical components and estimated three fixation indices: *F_CT_*, which can be interpreted as the relative divergence between HAT foci (Bipindi and Campo foci), *F_SC_*, corresponding to the relative divergence between populations within the Campo focus, and *F_ST_*, corresponding to the relative divergence among populations. *F_CT_* was tested by permuting populations among foci, *F_SC_* by permuting haplotypes among populations within a focus, and *F_ST_* by permuting haplotypes among populations among the foci. Moreover, pairwise *F_ST_* between populations were computed and their significance was tested by 10,000 permutations using ARLEQUIN.

The phylogenetic relationship among populations was assessed using DARwin (DARwin software http://darwin.cirad.fr/darwin). Genetic distances between populations were obtained by computing the usual Euclidian distance matrix based on haplotype frequencies. From this matrix, a dendrogram was constructed using the neighbor joining method (NJ) from Saitou and Nei [31] implemented in DARwin 5 (Perrier and Jacquemoud-Collet, 2006). The significance of each node was evaluated by bootstrapping data over a locus for 1000 replications of the original matrix.

To better understand the molecular relationships between intraspecific data, we connected haplotypes using a median-joining network [32] with the NETWORK 4.5.1.6. program (http://www.fluxus-engineering.com), equally weighting each locus, and setting the epsilon parameter to 20 to obtain a full median network. The median-joining network algorithm [32] combines the Kruskal algorithm for finding minimum spanning trees [33] and Farris's maximum-parsimony heuristic algorithm [34]. Moreover, to test for the impact of the allele phylogeny on genetic structuring, we estimated a global *N_ST_* and pairwise *N_ST_* between populations. The *N_ST_* statistic takes into account genetic distances between haplotypes [35], which we estimated as the sum of the squared differences of the repeat number at the microsatellite loci between two haplotypes [36]. If the differentiation follows a phylogeographic pattern and microsatellite markers a stepwise mutation model, *N_ST_* is expected to be larger than *G_ST_*. *N_ST_* were compared to *G_ST_* by permuting haplotypes in the genetic distance matrix between haplotypes, using the procedure implemented in the SPAGEDI program ([37]; 1000 permutations performed).

#### Geospatial analysis using GenGIS

GenGIS [38] was used to visualize haplotype diversity and its relationship between geographically distant populations.

## Results

### Genetic diversity in *S. glossinidius*

The complete dataset included multilocus genotypes for the 244 *S. glossinidius* strains from the 244 *G. palpalis palpalis* sampled in HAT foci in Cameroon (113 from Bipindi, 131 from Campo). The genome of these 244 *S. glossinidius* samples carried the four loci investigated in full length. The four microsatellite loci were polymorphic, and a total of 19 alleles were detected, ranging from three (ADNg 12/13) to six (ADNg 21/22, ADNg 15/16) alleles per locus (Table 1). Over all populations, the mean number of alleles was 2.25 for the ADNg 12/13 locus, 3.25 for the ADNg 5/2 locus, 3.75 alleles for the ADNg 21/22 locus, and 4.25 for the ADNg 15/16 locus (Table 3). The mean heterozygosity (*H*_E_) (Table 3) was quite different between loci, ranging from 0.07 (ADNg 12/13) to 0.59 (ADNg 15/16). Per population over the four loci, the heterozygosity (*H*_E_) varied from 0.35 to 0.41, corresponding to the villages Ebimimbang and Campo Beach/Ipono, respectively.

**Table 3:** Genetic diversity for four microsatellite loci and at the haplotype level in *Sodalis glossinidius* populations (standard errors are in parentheses).

	ADNg 21/22		ADNg 15/16		ADNg 12/13		ADNg 5/2		Mean over loci		Haplotypes		
Village (*N*)	*H*_E_	*Na*	*H*_E_	*Na*	*H*_E_	*Na*	*H*_E_	*Na*	*H*_E_	*Na*	*Nh*	*H*_R_	*H*_Eh_
Eb (113)	0.34	5	0.61	5	0.07	3	0.37	2	0.35 (0.22)	3.75 (1.50)	20	10.14	0.84 (0.02)
Ak (38)	0.15	2	0.59	3	0.05	2	0.71	4	0.38 (0.32)	2.75 (0.96)	12	10.56	0.91 (0.02)
CB/I (33)	0.18	4	0.63	5	0.12	2	0.70	4	0.41 (0.30)	3.75 (1.26)	15	14.00	0.92 (0.02)
Ma (60)	0.27	4	0.55	4	0.03	2	0.54	3	0.35 (0.25)	3.25 (0.96)	14	9.51	0.86 (0.02)
Mean	0.23 (0.09)	3.75 (1.26)	0.59 (0.03)	4.25 (0.96)	0.07 (0.04)	2.25 (0.50)	0.58 (0.16)	3.25 (0.96)			35^t^	11.05 (2.01)	0.87 (0.01)

Eb (Ebimimbang), Ak (Akak), CB/I (Campo Beach/Ipono), Ma (Mabiogo).

*N*, sample size; *H*_E_, heterozygosity; *Na*, number of alleles; *Nh*, number of haplotypes; *H*_R_, haplotypic richness;

t total; *H*_Eh_, haplotypic diversity.

The combination of the microsatellite alleles yielded a total of 35 haplotypes (Table 3). The four populations were polymorphic, showing 12–20 haplotypes. Their haplotypic diversities were 0.84, 0.86, 0.91, and 0.92 for Ebimimbang, Mabiogo, Akak, and Campo Beach/Ipono, respectively, with haplotypic richness of 10.14, 9.51, 10.56, and 14, respectively. The mean haplotypic diversity was 0.87.

### Population differentiation patterns

The population structure of *S. glossinidius* was explored at different hierarchical levels using AMOVA (Table 4). On haplotypic frequencies, AMOVA revealed that most of the variation was found among individuals within populations (97.8%). The fixation index reflecting the nested design of the samples indicated no overall differentiation between populations within the Campo focus (*F_SC_* = 0.003, *P* = 0.33) and a slight but not significant differentiation at the foci level (*F_CT_* = 0.019, *P* = 0.25). The genetic differentiation among the four populations was low (*F_ST_* = 0.022) but significant (*P* = 0.006). Pairwise population comparisons of genetic differentiation (*F_ST_*) are shown in Table 5. No differentiation was shown between the villages Akak and Campo Beach/Ipono (*F_ST_* = −0.005) nor between Campo Beach/Ipono and Mabiogo (*F_ST_* = −0.001). A positive but not significant differentiation (*F_ST_* = 0.009) was recorded between Mabiogo and Akak. Even though *F_ST_* values were relatively low, significant differences were shown between Ebimimbang and Mabiogo, Akak and Campo Beach/Ipono, with *F_ST_* of 0.018 (*P* = 0.023), 0.022 (*P* = 0.027), 0.032 (*P* = 0.014), respectively. This indicates a significant differentiation between the Bipindi (Ebimimbang) population and those of the three villages (Akak, Campo Beach/Ipono, and Mabiogo) in the Campo focus.

**Table 4:** Analysis of molecular variance (AMOVA) from haplotypic frequencies for *Sodalis glossinidius* microsatellite data.

Source of variation	d. f.	Variance components	Percentage of variation	Fixation indices / P-value
Among groups	1	0.009	1.93	0.019 (*F_CT_*)/0.252
Among populations within groups	2	0.001	0.29	0.003 (*F_SC_*)/0.333
Among all populations	240	0.432	97.78	0.022 (*F_ST_*)/0.006

Groups correspond to the two HAT foci, Bipindi (*S. glossinidius* population in Ebimimbang) and Campo (Campo Beach (CB)/Ipono, Akak, and Mabiogo populations). The three fixation indices are: *F_CT_*, which can be interpreted as the relative divergence between HAT foci, *F_SC_*, corresponding to the relative divergence between populations within the Campo focus, and *F_ST_*, corresponding to the relative divergence between populations.

**Table 5:** Genetic differentiation among *Sodalis glossinidius* populations based on *F_ST_* and *N_ST_* estimated from haplotype frequencies.

	Ebimimbang (*F_ST_*/*N_ST_*)	Akak (*F_ST_*/*N_ST_*)	Campo Beach/Ipono (*F_ST_*/*N_ST_*)
Akak	0.022*/0.008		
Campo Beach/Ipono	0.032*/−0.001	−0.005/0.022	
Mabiogo	0.018*/0.018	0.009/0.059	−0.001/−0.017

*P-value<0.05.

**Figure 1:**
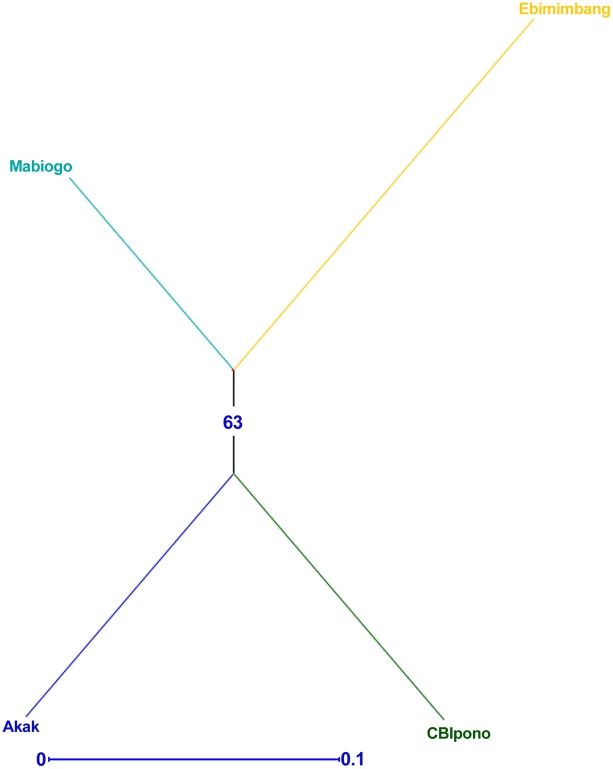
Neighbor-joining tree of populations of *Sodalis glossinidius*. The tree is based on calculation of a usual Euclidian distance using DARwin. Bootstrap probabilities are shown above the branch.

The neighbor-joining (NJ) tree calculated from the haplotype frequency using the usual Euclidian distance is shown in Figure 1. The NJ tree merges (1) the Ebimimbang *S. glossinidius* population (Bipindi HAT focus) with the Mabiogo population (Campo focus) and (2) the Akak and Campo Beach/Ipono populations (Campo focus), but the node is mildly supported by the bootstrap resampling (bootstrap value 63%).

### Haplotype network and distribution

**Figure 2:**
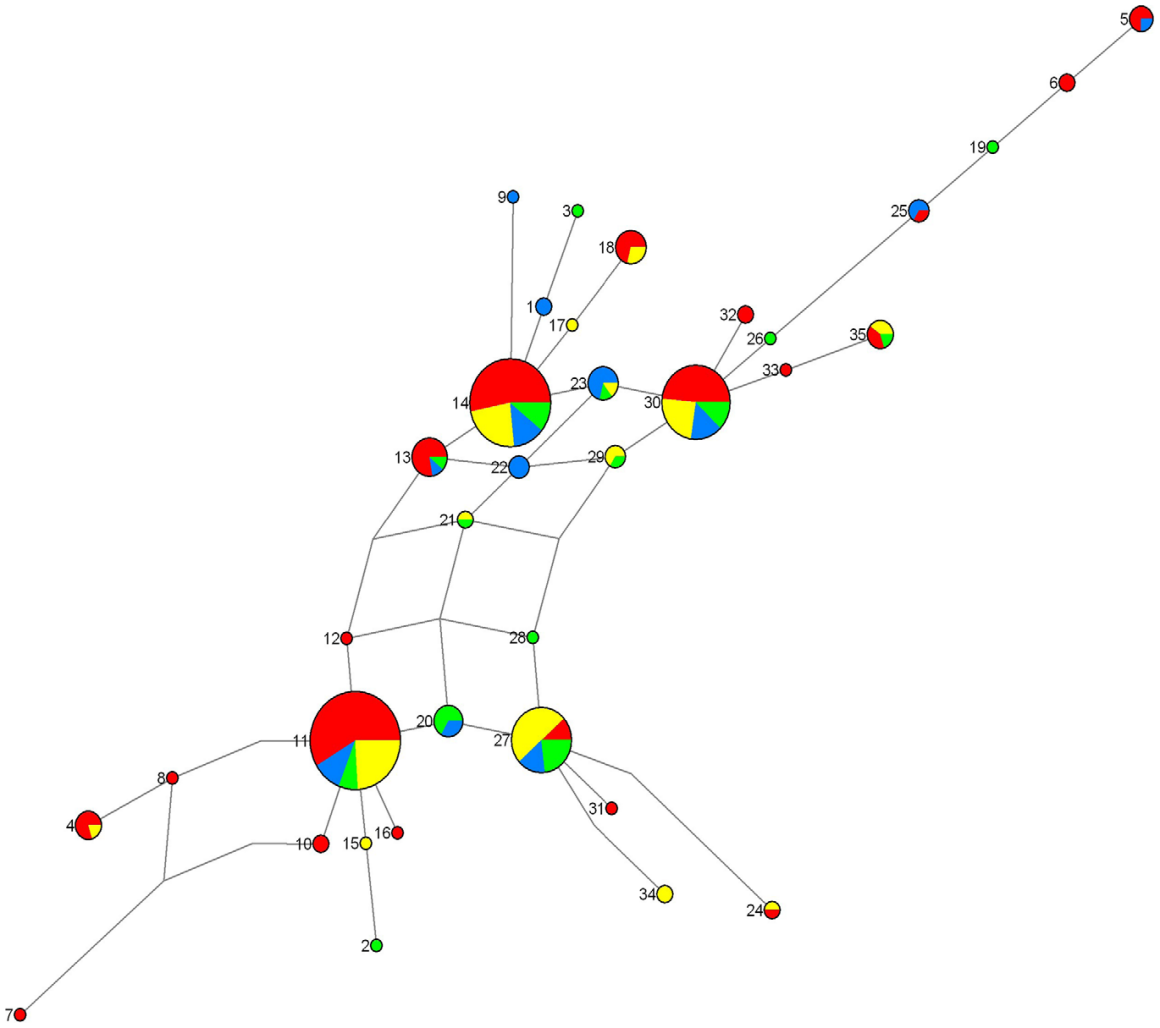
Median-joining network of *Sodalis glossinidius* haplotypes. The network was based on 35 haplotypes. Filled circles indicate the haplotypes, the numbers identify the haplotypes, with the size of each circle proportional to the observed frequency. The colors within the circles correspond to the different populations – red: Ebimimbang; blue: Akak; green: Campo Beach/Ipono; yellow: Mabiogo – and the size of the pie charts is proportional to the occurrence in the populations. Median vectors (mutation step not present in the sampled population) are indicated by dotted lines.

The four main haplotypes (H11, H14, H27, H30), showing overall frequencies above 0.05, were shared by the populations from the four sampling areas (Table S1 and Figure 2) and were present at high frequencies within the populations studied (except H27 in Ebimimbang, present at a low frequency). These haplotypes were separated by several mutation steps. The median-joining network resulted in a complex haplotype network and did not show a clear pattern of phylogeographic evolution (Figure 2). Global *N_ST_* was estimated at 0.015 and was not significantly different from global *G_ST_* (*P* = 0.72). Pairwise *N_ST_* between populations are shown in Table 5 and were not significantly different from *G_ST_*, but the *N_ST_* estimated between Akak and Mabiogo was almost significantly larger than *G_ST_* (*P* = 0.059).

**Figure 3:**
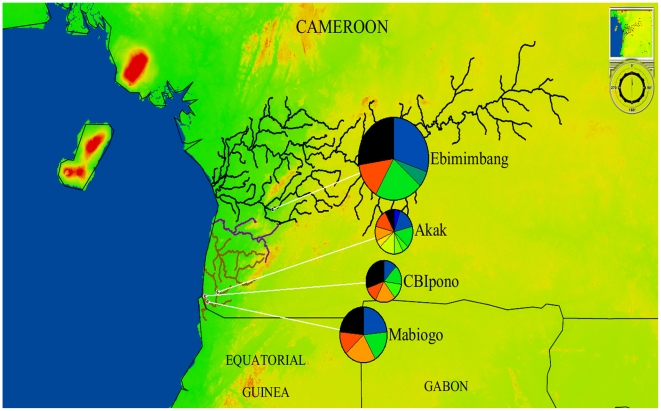
Georeferenced pattern of *Sodalis glossinidius* haplotype diversity. Map showing the geographic location of the *Sodalis glossinidius* sample sites, the pattern of haplotypic diversity, and the side basin. Each circle represents a population. The pie charts represent the frequencies of haplotypes within each population. Rare haplotypes (<5%) within the different sites are merged in black.

In addition, GenGIS was used to draw a georeferenced pattern of haplotype diversity (Figure 3). Figure 3 clearly shows that haplotypes with high frequencies are shared between populations. Moreover, the Akak and Campo Beach/Ipono populations displayed more haplotypes with frequencies above 0.05 (eight and five haplotypes, respectively) than Mabiogo (four haplotypes) and Ebimimbang (four haplotypes), reflecting the large genetic diversity of the first two populations.

## Discussion

The present study was conducted within our investigations on sleeping sickness and most particularly on the tripartite interactions between the vector (the tsetse fly), its secondary symbiont (*Sodalis glossinidius*), and the parasite (the trypanosomes) the vector transmits to humans and animals. This study was the first to take an interest in the population genetics of *S. glossinidius* in the field. Its aim was to analyze the genetic diversity of *S. glossinidius* populations from two human African trypanosomiasis foci, Bipindi (one village) and Campo (three villages), in South Cameroon, in order to detect possible differentiation / gene flow within or between the HAT foci. The *S. glossinidius* analyzed were those harbored by the flies (244 in total) sampled in the different areas of the two foci. Genotyping was performed using the variable-number-of-tandem-repeats (microsatellites) approach, not yet used to investigate *S. glossinidius* genetic diversity in the field. Four polymorphic loci were analyzed, the robustness and resolving power of this approach being maximized when large strain collections are analyzed using multiple loci [39], [40].

The microsatellite markers were polymorphic and had sufficiently high resolution to estimate the genetic structure of *S. glossinidius* isolated from *G. palpalis palpalis*. The populations, corresponding to the four villages analyzed, had high levels of genetic diversity, as indicated by the allelic richness and the proportion of heterozygotes (*H*_E_), whereas one locus (ADNg 12/13) was nearly nonpolymorphic. The combination of the four markers into haplotypes led to substantial overall diversity (*H*_Eh_ = 0.87). However, genetic diversity was lower in Mabiogo and Ebimimbang than in Akak and Campo Beach/Ipono. The lower genetic diversity of the Ebimimbang and Mabiogo *S. glossinidius* population may be associated with a lower effective population size in these villages. This could be due to the lower effective population size of its host, *G. palpalis palpalis*, or to the existence of a selective pressure exerted by the tsetse flies on the symbiont *S. glossinidius* in the populations concerned. It should be noted that a lower apparent fly density per trap and per day was observed in Ebimimbang in comparison with the other three villages, and particularly Akak [17], and in Mabiogo in comparison with Akak and Campo Beach/Ipono. However, this observation should be taken with caution because differences in the apparent fly density may not reflect differences in effective population sizes.

Within the Campo HAT focus, differentiation between populations was not significant. The village of Ebimimbang, located in the Bipindi HAT focus, showed significant *F_ST_* with the three villages in the Campo focus, with the foci 150 km apart and located on different river basins. The differentiation analysis, based on the pairwise *F_ST_* between populations and the AMOVA, revealed that the *S. glossinidius* populations presented a slight but significant differentiation between the Bipindi and Campo HAT foci.

The network and the georeferenced haplotype analysis showed that three frequent ancestral haplotypes were shared between the four populations and that there was not a geographic pattern of haplotypic diversity. These data suggest either that the gene exchange between populations occurred repeatedly or that the haplotypes derived from a common ancestral population.

The information provided by the *N_ST_* did not show an impact of the alleles' phylogeography on the structure of genetic diversity, except perhaps for the relation between haplotypes from the Mabiogo and Akak populations. However, the absence of information on the mutation process of microsatellite markers in *S. glossinidius* does not allow inferring the liability of *N_ST_*/*G_ST_* comparison in this species.

Finally, these results tend to show that the Akak and the Campo Beach/Ipono *S. glossinidius* populations may be considered as a single population, suggesting that gene flow occurred within the Campo HAT focus. Between Campo and Bipindi HAT foci, differentiation existed but was low. This could be explained by the fact that genes flow between the Ebimimbang population and the Campo focus is ongoing or has been maintained until recently at a level preventing strong differentiation.

As a symbiont of *Glossina*, with mainly a vertical transmission but also perhaps a horizontal transmission among matrilines of tsetse flies [41], the genetic diversity of *S. glossinidius* depends on its host. Our results suggest that gene flow exists between tsetse flies within the Campo HAT focus and that structuring may exist between the two foci, implying a limited gene flow, at least of female flies. The slight local differentiation among the *S. glossinidius* populations might be related to the fly migration rate between the HAT foci. The two HAT foci are located on different river basins (see Figure 3), but tsetse flies could move from place to place and form a continuous belt, which could be promoted by the presence of a large number of rivers and stream habitats, combined with suitable host availability allowing good dispersal conditions and a less confined spatial distribution of flies [42]. Finally, all these results suggest that the *S. glossinidius* populations of the two Cameroonian foci may be considered to belong to a lineage from which subgroups are genetically differentiating.

Genetic diversity was previously observed in *S. glossinidius* strains from insectary *Glossina palpalis gambiensis* species [15], [16] and was hypothesized to reflect differential host-driven selective pressures. In a previous study [17], sizeable differences between the sampled population of flies from Campo and Bipindi were recorded for the prevalence of *S. glossinidius* and trypanosome infections. Nevertheless, a significant association was found between the presence of *S. glossinidius* and the *Trypanosoma* infections of field populations of tsetse flies [17].

In conclusion, these results provide new information on the genetic diversity of *S. glossinidius* populations. They evidence the existence of differences between symbiont populations according to the flies' origin, the Campo or the Bipindi HAT focus. The evidence of a slight gene flow (or gene flow maintained up to very recently) between the two foci located about 150 km from each other was unexpected. This means that tsetse fly migration occurs despite this rather large distance. This finding is important in the context of sustainable vector control. Accurately estimating to what extent the genetic diversity of *S. glossinidius* populations depends on the population genetics of its host *G. palpalis palpalis* deserves to be studied: the genetic diversity analysis of tsetse fly populations will have to be undertaken within the same foci. Moreover, further investigations will consist in looking for a possible association between field tsetse fly infections by a given trypanosome species and the presence of *S. glossinidius*-specific haplotypes. These investigations could contribute to understanding the differences in the prevalence of *S. glossinidius* and trypanosomes between foci. The identification of *S. glossinidius* haplotypes potentially associated with vector competence could be included as diversity markers in epidemiological surveys, risk mapping and management, and vector control strategies.

## Supporting Information

Table S1 ***S. glossinidius*** **haplotypes found in populations from the four sampling areas: Ebimimbang (Bipindi focus), Akak, Campo Beach / Ipono and Mabiogo (Campo focus).**
(PDF)
