# Supplementary material for: Genetic Diversity and Population Structure of the Secondary Symbiont of Tsetse Flies, Sodalis glossinidius, in Sleeping Sickness Foci in Cameroon
Source: PLoS Negl Trop Dis. 2011 Aug 23;5(8):e1281. doi: 10.1371/journal.pntd.0001281 (PMC3160304; doi:10.1371/journal.pntd.0001281)
Supplement: Table S1 — S. glossinidius haplotypes found in populations from the four sampling areas: Ebimimbang (Bipindi focus), Akak, Campo Beach / Ipono and Mabiogo (Campo focus). (PDF) [file pntd.0001281.s001.pdf]

Populations *Haplotypes*	Akak (38)	CBIpono (33)	Ebimimbang (113)	Mabiogo (60)	Total
H1	2				2
H2		1			1
H3		1			1
H4			4	1	5
H5	1		3		4
H6			2		2
H7			1		1
H8			1		1
H9	1				1
H10			2		2
H11	6	4	34	14	58
H12			1		1
H13	1	1	7		9
H14	6	5	25	11	47
H15				1	1
H16			1		1
H17				1	1
H18			5	2	7
H19		1			1
H20	2	4			6
H21		1		1	2
H22	3				3
H23	5	1		1	7
H24			1	1	2
H25	2		1		3
H26		1			1
H27	4	6	3	13	26
H28		1			1
H29		1		2	3
H30	5	4	16	8	33
H31			1		1
H32			2		2
H33			1		1
H34				2	2
H35		1	2	2	5
